# Heart rate variability analysis using robust period detection

**DOI:** 10.1186/1475-925X-13-138

**Published:** 2014-09-23

**Authors:** Jørgen H Skotte, Jesper Kristiansen

**Affiliations:** National Research Centre for the Working Environment, Lersø Parkallé 105, DK-2100 Copenhagen, Denmark

**Keywords:** Heart rate variability, Robust periodogram, Power spectrum, HRV, FFT, RPD

## Abstract

**Objective:**

Heart rate variability (HRV) analysis, which is an important tool for activity assessment of the cardiac autonomic nervous system, very often includes the estimation of power spectra for series of interbeat intervals (IBI). Ectopic beats and artifacts have a destructive effect on the standard methods (Fourier transform, FFT) for frequency analysis. This study investigates an alternative method for calculation of the periodogram using a robust period detection (RPD).

**Method:**

Error free IBI series of 5 minutes for 221 subjects during one day were artificially distorted by randomly changing IBI values by ±15-40%. The low to high frequency rate (LF/HF) were calculated from periodograms estimated by the FFT, RPD and Lomb (LSP) methods for both error free and distorted series and for series with removed beats. Log transformed LF/HF values for series with distorted/removed beats were compared to undistorted values by linear regression.

**Results:**

For series with 10% of distorted IBI values the regression analysis between distorted and undistorted series showed a goodness of fit, coefficient and intercept of 0.98, 0.94 and 0.02, respectively. In comparison, the values of these parameters were (0.34, 0.46, -1.61) and (0.28, 0.42,-1.32) for the FFT and LSP methods, respectively. Similarly, the comparison between series with removed and undistorted beats yielded goodness of fit, coefficient and intercept of (0.98, 0.96, -0.01), (0.93, 0.78, -0.02) and (0.98, 0.95, 0.19) for RPD, FFT and LSP, respectively.

**Conclusion:**

The RPD method demonstrated superior performance compared to the FFT and LSP method by estimation of power spectral characteristics for HRV analysis.

**Electronic supplementary material:**

The online version of this article (doi:10.1186/1475-925X-13-138) contains supplementary material, which is available to authorized users.

## Background

Heart rate variability (HRV) is a method that is increasingly used to assess autonomic cardiac regulation of the heart in subjects during free-living conditions. Briefly, the beat-to-beat variation in heart rate (HR) is a result of the opposing influences of the parasympathetic and sympathetic divisions of the autonomic nervous system (see, for example, [1, 2]). Due to fact that it takes longer for cardiac pacemaker cells to respond to sympathetic neural signals compared to parasympathetic signals, the relative activity in these two divisions of the autonomic nervous system can be disentangled by analyzing the frequency content of the interbeat interval time series [3]. Thus, the high frequency (HF, 0.15-0.4 Hz) power in the interbeat interval series reflects parasympathetic influence on cardiac regulation, while the low frequency power (LF, 0.04-0.15 Hz) predominantly reflects sympathetic modulation of the cardiac rhythm. The ratio between the low and high frequency power (LF/HF) is interpreted as the balance between the sympathetic and parasympathetic modulation of cardiac rhythm [4–6], and is widely used because it captures essential physiological information in a single parameter [7–9].

Metrics for assessing HRV are generally based on either time-domain (time series) or frequency-domain analysis. Several advanced filtering techniques (linear and non-linear) have been described in the literature, but traditionally the fast Fourier transform algorithm (FFT) is a central part of the frequency-domain methods [10, 11]. Raw heart rate data consist of series of interbeat values (tachogram, distances between peaks in the QRS complex, RR data), which often contains errors or irregularities caused by artefacts or ectopic beats. Heart rate (HR) data recorded during everyday life including work hours often contain considerable amount of erroneous detected beats, typically during periods with intense movement. It is well known that the FFT analysis is highly sensitive to artefacts and even a small rate of faulty beats e.g. 1- 2% will cause bias in the calculation of the power spectrum [12, 13], hence it is mandatory to detect and remove artefacts. After the error correction process, the RR data must be interpolated and resampled at a fixed rate (e.g. 4 Hz). HRV data are typically calculated in time windows of 5 minutes and the FFT calculation can be applied once to this window or to a number of smaller sections (e.g. around 1 minute), for which the spectra afterwards are averaged (Welch’s method). Furthermore, it is normal to apply a weighting function (e.g. Hamming window) to the data before the FFT calculation to improve the resolution of the estimated spectrum. Consequently, the frequency analysis using FFT includes several steps with different methodological options. Another method for estimation of the power spectrum is the Lomb-Scargle periodogram (LSP), which unlike the FFT method, can estimate the power spectrum directly from the irregularly sampled RR data thus making the interpolation and resampling step unnecessary [14]. However, the LSP method is like the FFT method sensitive to outliers in the RR data [15]. Recently, a method has been described for robust period detection (RPD) of unevenly sampled data [16]. The method was developed for the analysis of periodicity in data from gene microarrays, but was expected to be useful for other types of uneven sampled biological data. To our knowledge this RPD method has not been used for HRV analysis.

The purpose of this study was to investigate the applicability of the RPD method to HRV analysis with special reference to HR data recorded during everyday life including considerable amount of faulty detected beats.

## Methods

By the RPD method spectral estimates were obtained in 5 minutes periods by the procedure described in ([16], Additional file 1). Briefly, the procedure includes the following steps:

From the actual (unevenly sampled) time of beats τ_1_,…,τ_N_, a new set of normalized indices t_1_,…,t_N_ are formed, where


Then a set of sine and cosine values are calculated by sin(2πkt_n_/N), cos(2πkt_n_/N) where k is a frequency index k = 0,1,…,N/2. These sine and cosine values are used as predictor variables for the measured interbeat values rr_1_,rr_2_,…,rr_n_ in a multilinear, robust regression model. From the regression coefficients A_sin,k_ and A_cos,k_, the power spectral estimate is calculated by


for the frequencies


where F_s_ is the mean sample frequency for the beat time series τ_1_,…,τ_N_.

The robust regression algorithm uses iteratively reweighted least squares with a bisquare weighting function, which assigns less weight to data points causing high residuals and zero weight for outliers (robustfit function called with the parameters Wfun = ‘bisquare’ and Tune = 4.6851, [17]). Two cycles of robust regression are executed. First, an initial run is carried out in which the spectral estimates are calculated according to the sequence k = 0,1,…,N/2. Then the coefficients of the initial spectrum are sorted according to magnitude and a second run of regression is carried out, where the frequencies are processed in the order of descending magnitude. In every step (frequency) of this run the residual from the preceding iteration is used as input i.e. the fitted sinusoidal of the preceding step is subtracted leaving the residual.

Spectral estimates were also obtained by calculation of the FFT and LSP periodograms [18]. The FFT periodogram was obtained by applying a linear interpolation and resampling with a sample frequency of 4 Hz. A Hamming window was applied to 5 minutes periods and the FFT algorithm processed for 1024 points. From the spectral estimates calculated by the RPD, FFT and LSP methods, the power was obtained in the low frequency range 0.04-0.15 Hz (LF) and the high frequency range 0.15-0.4 Hz (HF), after which the LF/HF ratio was calculated and logarithmic transformed.

The data used in this study included HR measurements obtained by the Actiheart monitor, which can record and store IBI values for 440.000 beats using two standard ECG electrodes adhered at the V1/V2 and V4/V5 positions, respectively. The beats are detected from signals with a sample frequency of 128 Hz, and IBI values are obtained with a resolution of 1 ms using an interpolation algorithm [19, 20].

IBI data were obtained for 221 subjects (92 females and 129 males in the age of 18–65 years), mainly blue collar workers, for one day of measurements including work hours, leisure time and sleep. The measurements were divided into 5 minutes periods and processed by an algorithm for detection of ectopic beats and artefacts. In the literature abnormal beats are typically defined as beats for which the IBI value deviate more than e.g. 20% of the previous normal beat [21]. In this study beats were classified as abnormal if IBI values deviated more than 15% (ectopic strength) from neighboring normal beats.

For error free 5 minutes periods a distortion procedure was carried out that randomly applied errors to beats (ectopic distortion) and groups of beats up to an error rate of 20%. The type of ectopic distortion applied was similar to the real ectopic beats and artifacts generally found in the recordings. A random number/set of single beats or all beats in a random cluster could be displaced with a random value in the interval ±15-40% or a beat could be excluded. Also, an ectopic distortion scheme was applied where all beats, which were selected for distortion, were just deleted from the IBI time series. Finally, a special distortion scheme was applied in which the randomly selected beats (as before) were displaced with exactly +15% or -15% in order to induce ectopic distortions just at the threshold level for error detection.

From the error free periods power spectra were estimated by RPD, FFT and LSP methods before and after applying the different distortion schemes. Then the logarithmic transformed LF/HF power ratios were calculated and comparisons made by means of linear regression between LF/HF values of the error free and distorted IBI series as a function of the distortion rate.

The very low frequency range (VLF: 0.003-0.04 Hz) were not included in the data comparison, so to increase the stationarity of the HR data most of the VLF frequencies were removed before calculation of the spectra. This was achieved by calculating the mean value of a 0.5 minute running window of the IBI values and subtracting the mean from every single IBI value.

By the calculation of spectral estimates it is an assumption that the data are stationary, which means that both the mean and variance should be approximately constant throughout the 5 minutes. To validate these assumptions a test for equal variance was done by splitting the 5 minute periods into 5 sections and performing a Brown-Forsythe test for equal variance on these sections [22]. All calculations were performed using the Matlab programing tool.

## Results

**Figure 1:**
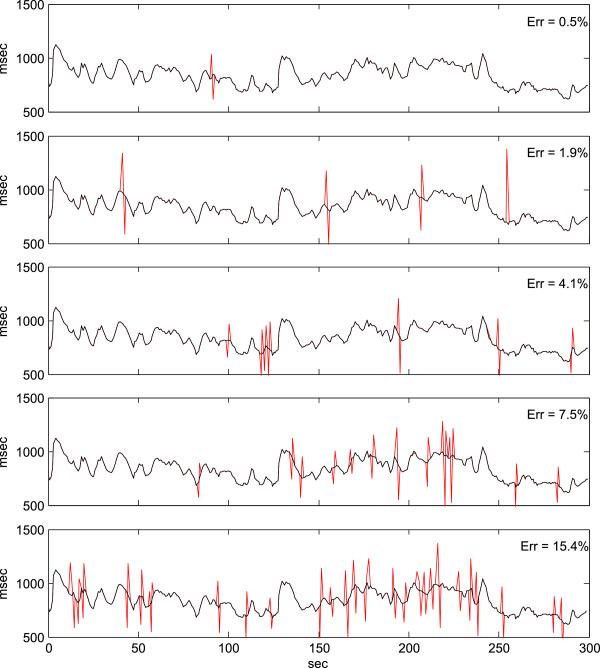
**Examples of increasing amount of distortion applied to a 5 minutes series of IBI values.** The series consists of 365 beats. The distortion rate increases from 0.5% in the uppermost graph to 15.4% in the bottom graph; red spikes represent distorted values.

**Figure 2:**
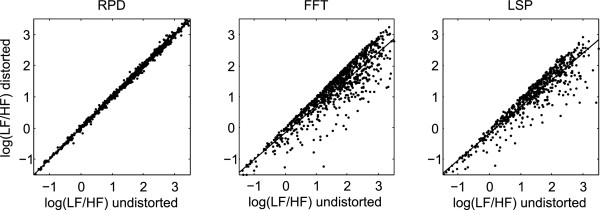
**Log transformed LF/HF ratios for distorted IBI series plotted versus undistorted series.** Power spectra were estimated by the RPD, FFT and LSP methods and log transformed LF/HF ratio calculated for 904 five minutes periods with a distortion rate between 0.5% and 1.5%. Regression for RPD: log(Y) = 0.996∙log(X) + 0.002, R^2^ = 0.998; regression for FFT: log(Y) = 0.755∙log(X)-0.364, R^2^ = 0.582; regression for LSP: log(Y) = 0.738∙log(X)-0.520, R^2^ = 0.603.

The data set consisted of all 5 minutes periods from 221 subjects monitored for HR during 1 day. Approximately 50% of the 5 minutes periods were without abnormal beats according to the criterion that normal IBI intervals should not deviate more than 15% from its neighboring intervals. Thirty percent of the 5 minutes periods were found to include errors but with an amount less than 10%. Figure 1 shows an example of a 5 minutes period without abnormal beats that has been processed by the ectopic distortion algorithm to induce random errors of varying ectopic strength.Figure 2 shows a plot of the LF/HF ratio of power spectra estimated from IBI series where approximately 1% of the IBIs have been changed corresponding to a displacement of the beats with a distance of ±15-40% to its neighbors. The LF/HF ratio for the ectopic distorted IBI series are compared with the undistorted series calculated by the RPD, FFT and LSP methods. It is evident that the small amount of distorted beats (typically 1–3 beats in an 5 minutes period, 1% of distorted IBIs corresponds to 0.5% distorted beats) have a large influence on the FFT and LSP calculated periodogram and very little influence on the RPD results.

**Figure 3:**
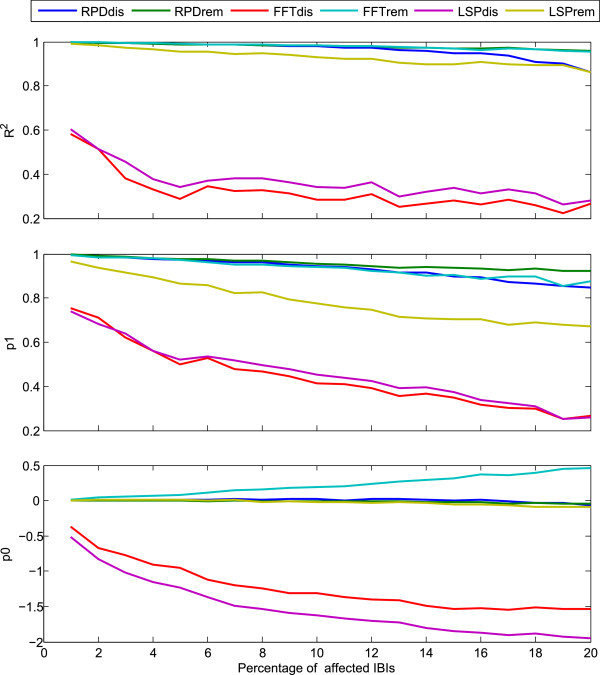
**Regression between LF/HF ratios for IBI series with distorted/removed beats and undistorted series.** Linear regression log(Y) = p1∙log(X) + p0 was calculated for log transformed LF/HF ratios for IBI series with distorted/removed beats (Y) and undistorted series (X) by the RPD, LSP and FFT methods (distortion rates up to 20%). Top: goodness of fit R^2^; Middle: coefficient p1; Bottom: intercept p0 (dis: IBI series with distorted series, rem: IBI series with removed beats).

**Figure 4:**
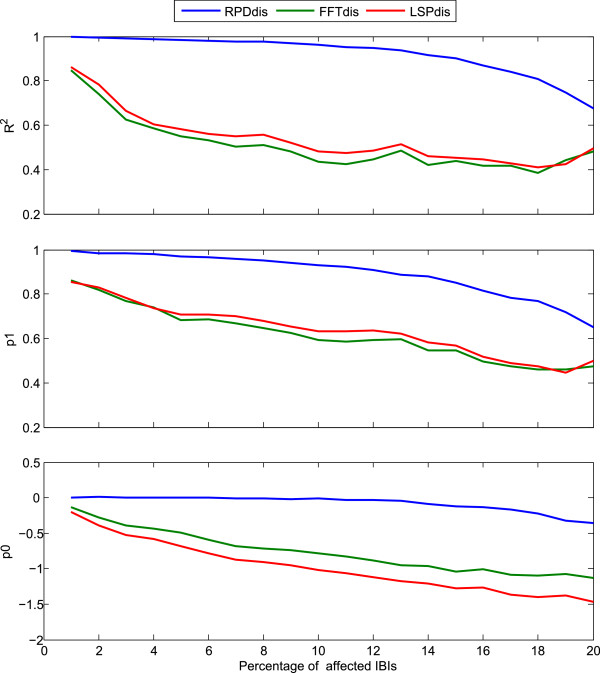
**Comparison between the methods RPD, FFT and LSP for series ectopically distorted ±15%.** Results of linear regression log(Y) = p1∙log(X) + p0 between log transformed LF/HF ratios for IBI series with beats ectopically distorted with a strength of precisely ±15% (Y) compared to undistorted series (X) for the RPD, FFT and LSP methods (distortion rates up to 20%). Top: goodness of fit R^2^; Middle: coefficient p1; Bottom: intercept p0.

Figure 3 shows the results of standard, linear regression between log transformed LF/HF ratios for IBI series with ectopic distorted beats (ectopic strength ±15-40%) and removed beats with distortion rates up to 20% compared to the undistorted series for the three different methods. For a distortion rate of 20% the RPD method shows a decrease in goodness of fit (R^2^) to 0.96 if the distorted beats are removed and to approximately 0.90 if the distorted beats are included in the calculation. Similarly, there is a decrease in the regression coefficient p1 to 0.93 and 0.87, respectively; however, the intercepts p0 are very close to zero. The LSP method demonstrates slightly lower R^2^ values and considerably lower coefficient values than the RPD methods when the ectopic beats were removed. There are no differences in R^2^ values between the RPD and FFT method if the distorted beats are removed and the spectra are estimated from the remaining IBIs; also the coefficient values for the FFT method approximate those of the RPD methods, however, the intercept values are increasing steadily. For the FFT and LSP method both the goodness of fit and regression coefficients are considerable below 1.0 and the intercept values below -0.5, when ectopic beats are included in the analysis. Table 1 lists the regression parameters for a distortion rate of 10% read from Figure 3, and some examples of the deviations between the methods calculated for selected LF/HF values in the range 0.1-10 are shown in Table 2.The results obtained when the ectopic distortion strength is precisely ±15%, are shown if Figure 4. It appears that the FFT and LSP methods are strongly affected by this level of distortion though there is some improvement compared to the results for ectopic distortions beyond ±15%. However, the RPD method performs well for distortion strengths of ±15% for error rates up to approximately 10%.

**Table 1:** **Results of linear regression between LF/HF ratios for IBI series with distorted/removed beats and undistorted series**

Regression parameter	RPDdis	RPDrem	LSPdis	LSPrem	FFTdis	FFTrem
R^2^	0.98	0.98	0.34	0.93	0.28	0.98
p1	0.94	0.96	0.46	0.78	0.42	0.95
p0	0.02	-0.01	-1.61	-0.02	-1.32	0.19

Regression parameter values for the linear regression log(Y) = p1∙log(X) + p0 between log transformed LF/HF ratios for IBI series with distorted/removed beats (Y) and undistorted series (X) by the RPD, LSP and FFT methods. The distortion rate was 10%. ‘dis’ denotes series with distorted beats and ‘rem’ denotes series with removed beats.

**Table 2:** **Examples of differences (percent) of LF/HF ratios for IBI series with distorted/removed beats compared to undistorted series**

	LF/HF differences for series with distorted/removed beats					
LF/HF for undistorted series	RPDdis	RPDrem	LSPdis	LSPrem	FFTdis	FFTrem
0.1	17%	9%	-31%	63%	2%	36%
0.3	10%	4%	-62%	28%	-46%	28%
1	2%	-1%	-80%	-2%	-73%	21%
3	-4%	-5%	-89%	-23%	-86%	14%
10	-11%	-10%	-94%	-41%	-93%	8%

The differences are calculated by the regression parameter values p1 and p0 in Table 1. The distortion rate was 10%. ‘dis’ denotes series with distorted beats and ‘rem’ denotes series with removed beats.

A total of 29359 five minutes periods were found to be error free. In 81% of these periods the hypothesis of equal variance throughout the period was rejected (p = 0.05), and in only 0.4% of the periods equal variance was found (p = 0.95). For the periods with the equal variance the regression between log transformed LF/HF ratios for FFT and RPD periodograms showed a R^2^ value of 0.96 and the coefficients (p0,p1) = (0.28,0.93), while for the periods without equal variance R^2^ = 0.79 and (p0,p1) = (0.47,0.83). Similar regression calculations for stationary periods of log transformed LF/HF ratios for LSP and RPD periodograms showed a R^2^ value of 0.98 and (p0,p1) = (-0.02,0.95), while for the periods without equal variance R^2^ = 0.89 and (p0,p1) = (0.26,0.83).

## Discussion

This study compared spectral estimates obtained by a new method using a robust period detection to the traditional FFT method and the Lomb-Scargle method. Error free 5 minutes periods of heart rate data were artificially distorted by displacing randomly selected beats by ±15-40%. Periodograms were estimated by all three methods for the basal error free periods, periods with distorted beats and periods with the distorted beats removed. The RPD method demonstrated good performance compared to the FFT and LSP method when calculating the low to high frequency ratio of the periodogram. For example, when using the RPD method the LF/HF estimates are almost identical for error-free periodograms and for periodograms with an error rate of 10% (of IBI values). RPD with removing abnormal beats resulted in deviations from +9% to -10% for LF/HF ratios in the range 0.1-10, and without removing abnormal beats, deviations were from +17% to -11% (Table 2). Similarly, for the FFT method with removed (and linear interpolated) abnormal beats, the deviations were from +36% to +5%. The occurrence of abnormal beats in heart rate data for periodogram estimation by the FFT and LSP methods result in the well-known underestimation of the LF/HF ratio, because of the high levels of HF power associated with peaks in the tachogram [22]. The deviation for the LSP method with removed abnormal beats (10% error rate) was in the range +63% to -41%, which is higher than expected as the LSP method is reported to be superior to the FFT method [23]. The reason for the poorer agreement of the LSP method is not known, but it could be speculated that the data of this study include real heart rate recordings, for which the requirement of stationarity very often cannot be fulfilled, while other studies of the LSP method mainly used simulated, stationary heart rate data. For higher error rates the RPD method with removing the abnormal beat performs better than the RPD method without removing the abnormal beats. For example for a an error rate of 20% the RPD method with removing abnormal beats yield deviations of +13% to -18% ((p0,p1) = (-0.04,0.93)), while without removing the abnormal beats, the deviation are +32% to -34% ((p0,p1) = (-0.07,0.85)).

For error rates up to approximately 10% the RPD method performed well for an ectopic distortion equal to ±15%, while the FFT and LSP were strongly affected. Beats with an ectopic distortion of ±15%, will just remain undetected by the procedure for finding and removing abnormal beats and artifacts, and these faulty beats have a large impact on the FFT and LSP methods. This clearly demonstrates the robustness and benefit of the RPD method compared to the FFT and LSP method, since no procedure for removing faulty beats is perfect; generally, there will be a tradeoff between detection of greatest possible number of abnormal beats and not to eliminate too many normal beats.

In theory the calculation of spectral estimates (and correlation functions) are based on the assumption of stationary data, which means that mean and variance do not vary significantly (weak stationarity). The applied 0.5 minutes mean subtracting procedure will to some degree ensure that the mean do not vary significantly, so the stationarity would mainly depend on approximately equal variance throughout the 5 minutes period. Only very few periods (0.4%) were found to meet an equal variance criteria set up in this study and accordingly to show an approximate stationarity. There was a better agreement between the FFT and RPD methods for these periods (R^2^ = 0.96, (p0,p1) = (0.28,0.93)) than between periods without equal variance and accordingly non-stationary (R^2^ = 0.79, (p0,p1) = (0.47,0.83)). The regression result for the stationary periods corresponds to an overestimation of the LF/HF ratio by 13% to 55% for ratios between 0.1 and 10 for the FFT method compared to the RPD method. This supports the validity of the present RPD method, since it reported that the FFT method with linear resampling can overestimate the LF/HF ratio by 50% [22]. A similar comparison between the LSP and RPD method for the stationary periods yielded LF/HF ratios for the LSP method to be within +10% to -14% of the RPD LF/HF ratio in the range 0.1 to 10 (R^2^ = 0.98, (p0,p1) = (-0.02,0.95)), so this shows a good agreement supporting the validity of the RPD method. However, for non-stationary recordings the same differences between the methods were from +92% to -12% (R^2^ = 0.89, (p0,p1) = (0.26,0.83)). The poorer agreement between the methods for the non-stationary periods is not surprising because of the violated stationarity assumption. We have included all periods both stationary and non-stationary in the calculations presented in Figures 2 and 3, since practical studies reported in the literature rarely elaborate on this issue.

One drawback of the RPD method is the very time consuming calculations of the periodogram compared to the LSP and FFT methods; the calculation of a periodogram for a 5 minutes period takes around 1 sec, 40 msec and 1 msec for the RPD, LSP and FFT methods, respectively, executed as a Matlab script by a standard PC. However, for most offline applications this might not be an issue.

## Conclusion

The RPD method demonstrated a superior capacity for estimation of the low to high frequency power ratio of the periodograms for heart rate data with ectopic beats and artifacts compared to the FFT and LSP method. Especially, a few marginally ectopic beats typically not recognized by error detecting procedures, have very little influence on the RPD result while seriously affecting the FFT and LSP methods. For error free and stationary heart rate data the RPD showed results similar to the LSP method, and to FFT method, when taking into account the inherent low-pass filtering characteristic of the FFT method.

## Electronic supplementary material

Additional file 1: **Matlab code for robust period detection.** (ZIP 113 KB)

## Abbreviations

RPD: Robust periodic detection
IBI: Interbeat interval
HRV: Heart rate variability
FFT: Fast fourier transform
LSP: Lomb-scargle periodogram
HF: High frequency
LF: Low frequency
VLF: Very low frequency
dis: Distorted
rem: Removed.
