# Supplementary material for: Heart rate variability analysis using robust period detection
Source: Biomed Eng Online. 2014 Sep 23;13:138. doi: 10.1186/1475-925X-13-138 (PMC4228159; doi:10.1186/1475-925X-13-138)
Supplement: Supplementary file 1 — Additional file 1: Matlab code for robust period detection. (ZIP 113 KB) [file 12938_2014_884_MOESM1_ESM.zip › index.html/Readme.pdf]

Supplementary materials for

Skotte JH, Kristiansen J:
Heart rate variability analysis using robust period detection.
BioMedical Engineering OnLine 2014, 13:138

The 4 Matlab text files

- *fitSp_corr.m*
- *helpScript.m*
- *oneFrequencyTest.m*
- *pvalues.m*

are supplementary materials for

Ahdesmäki M, Lähdesmäki H, Gracey A, Shmulevich I, Yli-Harja O:
Robust regression for periodicity detection in non-uniformly sampled time-course gene expression data.
BMC Bioinformatics 2007, 8:233.

These algorithms are also available as a R package in the CRAN project (http://www.r-project.org).

The Matlab text file

- *fitSp_corr_1.m*

contains the function actually used by the present study to calculate the robust spectral estimates,and it includes a few minor changes to the function fitSp_corr.m
